# Applications of Microct Imaging to Archaeobotanical Research

**DOI:** 10.1007/s10816-023-09610-z

**Published:** 2023-05-29

**Authors:** Aleese Barron

**Affiliations:** 1 School of Archaeology and Anthropology, Australian National University, Banks Building, Canberra, Canberra ACT 2601 Australia; 2 Department of Materials Physics, Research School of Physics, Australian National University, Canberra, Canberra ACT 2601 Australia

**Keywords:** microCT, Plant domestication, 3D visualization, Archaeological parenchyma, Pottery inclusions, Underground storage organs

## Abstract

The potential applications of microCT scanning in the field of archaeobotany are only just beginning to be explored. The imaging technique can extract new archaeobotanical information from existing archaeobotanical collections as well as create new archaeobotanical assemblages within ancient ceramics and other artefact types. The technique could aid in answering archaeobotanical questions about the early histories of some of the world’s most important food crops from geographical regions with amongst the poorest rates of archaeobotanical preservation and where ancient plant exploitation remains poorly understood. This paper reviews current uses of microCT imaging in the investigation of archaeobotanical questions, as well as in cognate fields of geosciences, geoarchaeology, botany and palaeobotany. The technique has to date been used in a small number of novel methodological studies to extract internal anatomical morphologies and three-dimensional quantitative data from a range of food crops, which includes sexually-propagated cereals and legumes, and asexually-propagated underground storage organs (USOs). The large three-dimensional, digital datasets produced by microCT scanning have been shown to aid in taxonomic identification of archaeobotanical specimens, as well as robustly assess domestication status. In the future, as scanning technology, computer processing power and data storage capacities continue to improve, the possible applications of microCT scanning to archaeobotanical studies will only increase with the development of machine and deep learning networks enabling the automation of analyses of large archaeobotanical assemblages.

## Introduction

Archaeobotanical evidence of early human-mediated exploitation, transportation and alteration of some of the world’s most important crop species is limited. Crop species from geographical regions with low archaeobotanical sampling rates and/or that produce types of botanical evidence with low archaeological visibility are currently underrepresented in the literature (Paz, 2005; Denham et al., 2009; Castillo & Fuller, 20,120; Bishop, 2021; Bishop et al., 2022). These include sexually-propagated cereal and legume species and asexually-propagated fleshy underground storage organs (USOs). In some cases, archaeobotanists are currently reliant on the fields of genomics and historical linguistics rather than direct archaeobotanical evidence to fill in major gaps relating to the histories of some major food crops (Diamond & Bellwood, 2003; Lebot, 2009; Mikic´, A., Medovic´, A., Jovanovic´, Z., & Stanisavljevic’, N. 2014; Denham et al., 2020). While new excavations and the consistent application of current archaeobotanical techniques are crucial to helping fill in both geographical and chronological gaps, the development of new archaeobotanical techniques, the extraction of new types of archaeobotanical material as well as the reanalysis of previously excavated material should be undertaken to increase both the quantity and quality of currently available archaeobotanical evidence.

An emerging imaging and analytical technique currently underutilized in archaeobotany is microCT scanning. The technique is a powerful tool for extracting large amounts of three-dimensional information from archaeological samples quickly and non-destructively, which can be digitally rendered, manipulated and analysed as well as easily shared or stored for prosperity. While microCT has found many novel uses within the field of archaeology, its application to imaging archaeobotanical materials was until recently very limited. Within the last ten years, a handful of studies have presented novel ways in which microCT can be utilized to answer archaeobotanical questions, however, the technique is yet to be applied systematically to analyse large numbers of samples consistently across whole assemblages. The increasing access researchers have to lab-based microCT systems coupled with the development of microCT visualisation protocols specifically designed for imaging and analysing archaeobotanical materials (e.g. Barron & Denham, 2018) could see the technique become regularly employed as part of the wider suite of archaeobotanical analytical methods. In this paper, I will outline the technique’s history of use in the fields of archaeology and archaeobotany, as well as the closely related fields of geoscience, botany and palaeobotany, before outlining possible future methodological developments and potential applications to archaeobotany.

## Computed Tomography

X-ray computed tomography was originally developed in the 1970s (Ambrose, 1976; Beckmann, 2006; Hounsfield, 1973; Ommaya et al., 1976) and initiallly used for medical imaging (﻿Ledley et al., 1974; ﻿Paxton & Ambrose, 1974). As the technique has been refined, scanning technology improved and computing power increased exponentially over time (see Kalender, 2006 for review), it has come to be used in an extremely wide range of fields, including biology, geology, materials science, palaeontology and biomedical engineering (see Staedler et al., 2013, Table S1). X-ray computed tomography is essentially the process of taking multiple 2D X-rays, or projections, from different orientations around an object and reconstructing those projections to form a digital, three-dimensional model of the object (see Brooks & Chiro, 1976; Kak & Slaney, 1988). While X-rays are the most commonly used type of radiation for CT imaging, neutrons have also been utilized to image archaeological samples based on comparable principles (see De Beer et al., 2009; Jacobson et al., 2011; Bertrand et al., 2012; Saprykina et al., 2019). X-ray images are formed by differential absorption, deflection and transmission of penetrative X-rays through an object. X-ray attenuation is affected by both the composition, density and thickness of the material and thus provides information regarding morphological form and changes, both internally and externally; however, it can only provide relative compositional information based on comparative attenuation values of assumed or known materials (see Kak & Slaney, 1988; Hsieh, 2015; Stock, 2020 for technique overviews). Therefore, X-ray CT is ideal for imaging heterogeneous samples comprised of materials with vastly different attenuation values. The two-dimensional x-ray projections produced during scanning are processed using dedicated reconstruction algorithms to produce a three-dimensional model, which is essentially a three-dimensional map of X-ray attenuation values at all points throughout a scanned object (see Willemink and Noël 2019 for evolution of image reconstruction methods).

### MicroCT

MicroCT was first developed in the 1980s (Elliott & Dover, 1982, 1985; Feldkamp & Jesion, 1986; Flannery et al., 1987; Sato et al., 1981; Suzuki et al., 1988) as a higher resolution and higher energy type of CT-imaging with no definitive differentiation between traditional CT and microCT. The basic setup of microCT systems differs from medical CT scanners with the sample rotating between a stationary X-ray source and detector for microCT, as opposed to the sample/patient remaining stationary while the X-ray source and detector rotate around them in a medical scanner (see Flannery et al. 1987 Fig. 2 for basic source-detector schematic). X-ray radiation dosage and exposure are severely limited for the health of patients in medical imaging, whereas this is of little importance for imaging archaeobotanical samples using microCT. However, the impact scanning has on aDNA, isotope ratios, and OSL dating remains largely unknown (see Immel et al., 2016; Wanek and Rühli 2016; Sears et al., 2016; Murphy et al., 2019).

There are two major types of microCT systems widely in use, lab-based and synchrotron, which differ by their X-ray sources, and, therefore, the types of X-ray beams. Lab-based microCT systems generally use tube X-ray transmitters, which produce polychromatic beams (Sasov and Dyck 1998). Synchrotron microCT systems use synchrotron light sources which produce tunable, monochromatic beams of higher flux and intensity enabling smaller spot sizes resulting in higher image resolution (Grodzins, 1983a, b; Thompson et al., 1984; Flannery et al., 1987; Baruchel et al., 2006). Generally, synchrotron high-resolution X-ray microCT is carried out at large facilities with a synchrotron radiation source, such as a particle accelerator, making the technique significantly more difficult to access and beam time is severely limited compared to lab-based microCT systems.

There are currently no standard protocols for the scanning of archaeobotanical samples, or any other type of samples, so a large number of parameters are determined by the user, dependent on the size and composition of samples and research questions of interest. These parameters include tube voltage and current, exposure time, step size, beam spot size, and the use of any physical filters. The variety of microCT systems, flexibility of scan parameters as well as the subjectivity of subsequent visualization and segmentation methods make it currently difficult to reliably compare data and analytical results obtained using different microCT systems (Baveye et al., 2010).

**Fig. 1:**
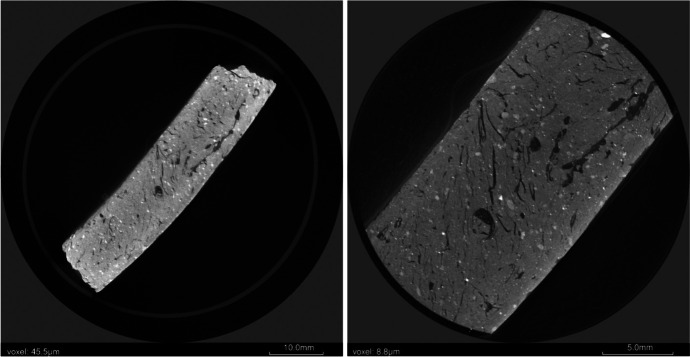
2D radiographic slices of sorghum-tempered pottery sherd. Left. Slice from full sherd scan. Right. Slice from ROI (region of interest scan

Image resolution (voxel size) is inversely proportional to sample size, specifically sample diameter, meaning that images of large samples may not possess the level of detail required for desired results. In fields such as geology, this limit of voxel size is often overcome by simply extracting subsamples using destructive coring methods, however, this option is not ideal for archaeological samples. The limitation on voxel size can be non-destructively overcome by the implementation of a region of interest (ROI) scan (Chityala et al., 2004; Kyrieleis et al., 2011) in addition to a full object scan. This process produces a higher resolution scan of a portion of the sample, yet extracts the subsample non-destructively (see Fig. 1).

### Sample preparation

One of the major reasons that microCT scanning has found widespread use in archaeology is the non-destructive nature of both sample preparation and image processing. When mounting a sample for microCT scanning the primary concerns are to secure it to the stage and stabilize all parts of the sample so that no movement occurs during or between radiographs. If all or part of a sample moves during scanning it can lead to blurred 3D images, particularly around the sample edges, after digital reconstruction. Samples are often mounted in any kind of vessel, ideally a tube composed of a low attenuation material, and stabilized inside using packing material (of significantly lower density than the sample if possible). However, sample mounting is dependent on the shape and nature of the sample and the technical specifications of the CT system (see Du Plessis et al., 2017).

**Fig. 2:**
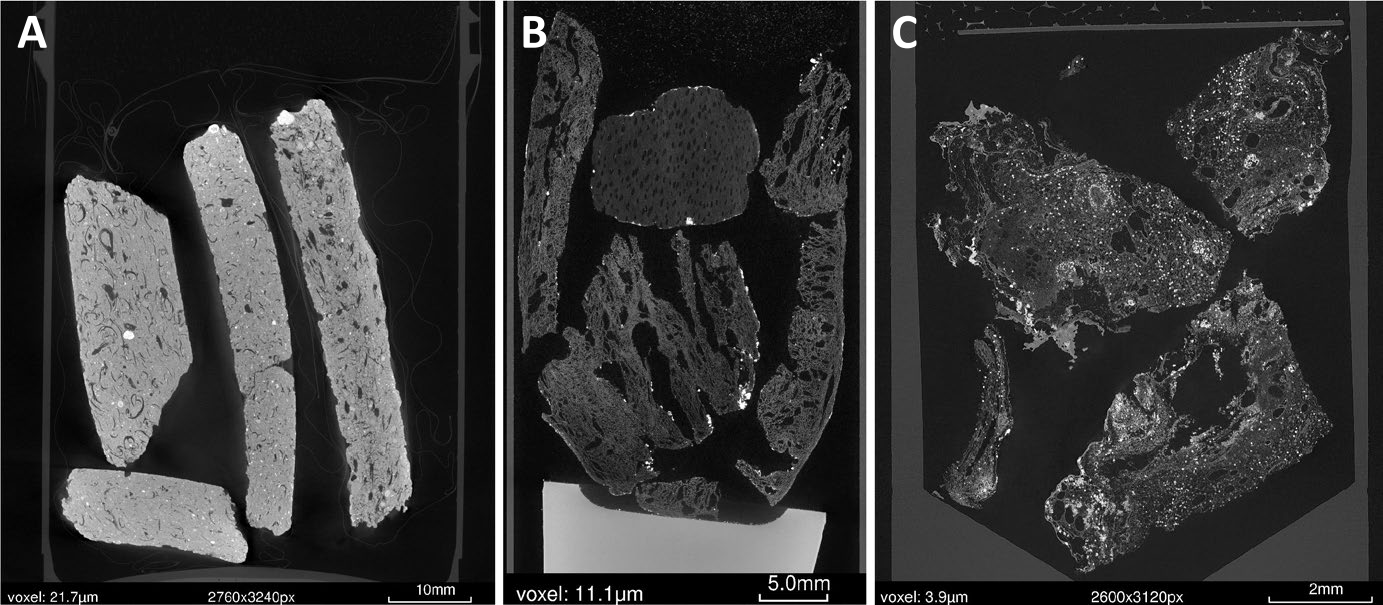
2D x-ray slices of samples prepared for tomographic imaging. A. African pottery samples (3 tempered with sorghum on the left and 1 tempered with pearl millet on the right) still in plastic sample bags and stacked inside a plastic specimen jar. B. Archaeobotanical samples (five charred parenchyma samples and one wood charcoal) stacked inside a plastic tube and secured from above with packing foam. C. Charred *Colocasia esculenta* reference sample stacked inside a plastic tube and secured from above with a plastic disc (to aid with post-scanning segmentation) and packing foam

Figure 2 shows examples of sample preparation and stabilization methods used for the scanning of different types of archaeobotanical samples. All samples are mounted in plastic tubes and the organic-tempered pottery sherds in Fig. 2a are imaged within their plastic sample bags. This was done to avoid separating sherds from the contextual information written on the plastic bags, which attenuate significantly less X-rays than the sherds and thus do not affect the imaging of the sherds themselves. The pottery sherds in Fig. 2A were able to be stabilized by their own rigidity against the walls of the tube whereas the wood and parenchyma sample in Figs. 2B & C were weighted down by packing foam. The taro fragments in Fig. 2C were also separated from the foam by a sheet of stiff plastic to create a clear separation between the sample and foam to enable simpler digital segmentation for visualization and/or analysis.

### Visualisation and Analysis

**Fig. 3:**
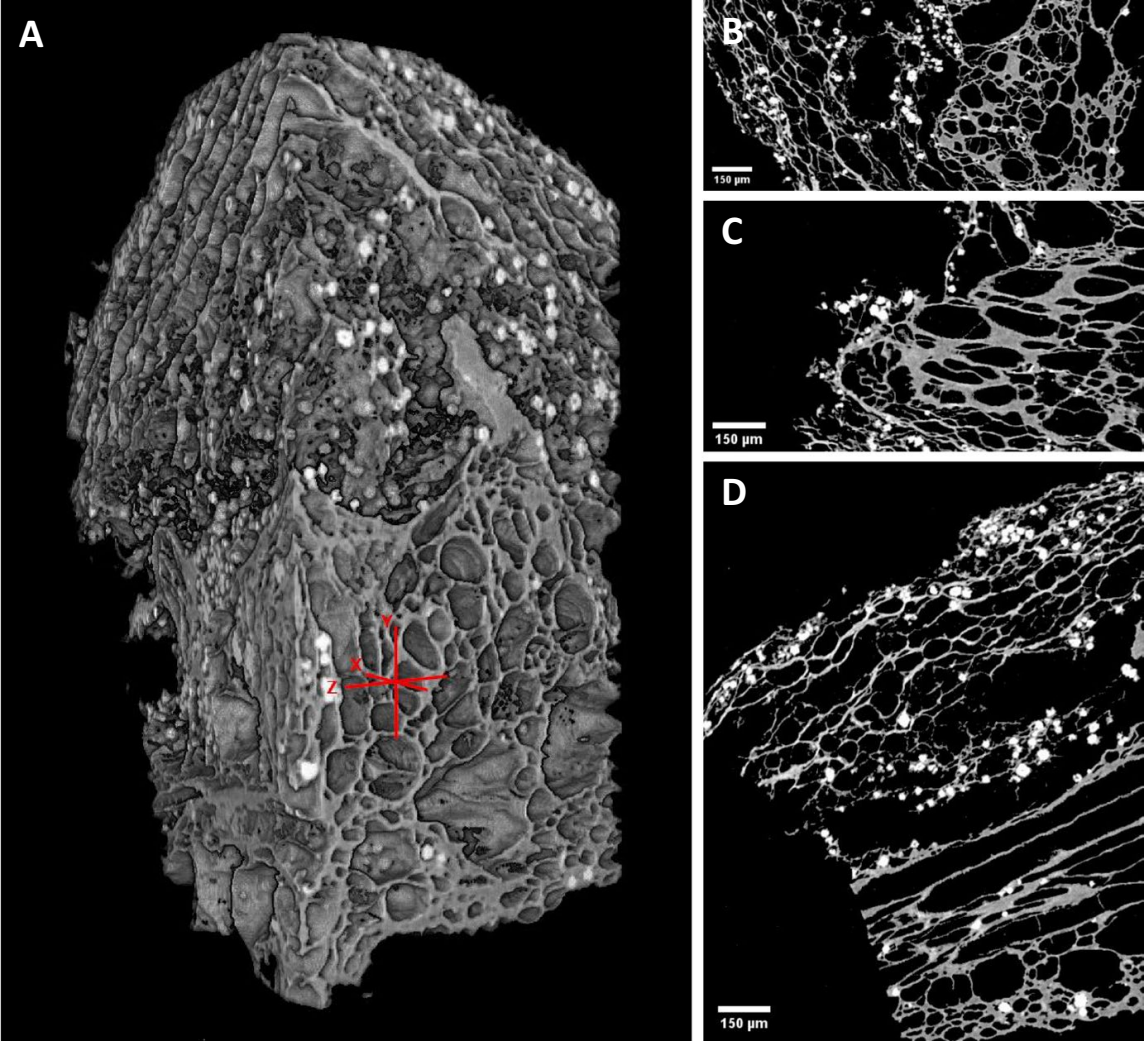
3D rendering of sweet potato (*Ipomoea batatas*) reference sample and views of 2D tomographic slices in Drishti Paint. A. 3D rendering with orange crosshairs indicating the location of slices C-D. B. 2D view of slice along x-axis. C. 2D view of slice along y-axis. D. 2D view of slice along z-axis

The digital datasets produced by microCT scanning can be used for visualization or analytical purposes and can be rendered, segmented and/or animated to access internal or external morphologies. The data can be viewed by two-dimensional slice from any direction or as a three-dimensional model, providing perspectives from infinitely more angles than two-dimensional imaging techniques (Fig. 3). MicroCT data can be segmented (i.e. partitioned and labelled) based on various properties such as X-ray attenuation value, shape, size and connectedness and each segment can be visualized and/or analysed individually. Many 3D image processing software packages are commercially available such as Avizo, VG Studio Max and Dragonfly, whereas others are open-source, such as Drishti (Hu et al., 2020; Limaye, 2012). Many of these software packages offer both quantitative and qualitative analytical methods with the abilities to alter visual properties—such as rendering colours, lighting, opacity and camera orientation—as well as to calculate quantitative characteristics—such as volume, distribution and compositional ratios (see Maire & Withers, 2013).

The digital nature of microCT datasets makes them easy to share virtually with researchers around the globe (see Stuppy et al., 2003; Tuniz et al., 2013). This is particularly useful for the study of rare and/or valuable archaeobotanical samples, which are often too fragile to be transported or are not legally allowed to be transported outside of their country of origin (Calo et al., 2019). Digital datasets can also reduce the need for researchers to undertake expensive and, in the current public health and climatic crises, increasingly difficult travel to access archaeological collections.

The ability to share microCT datasets is only limited by the size of the datasets and the limited capacity of existing file sharing platforms. Online repositories, often hosted by university-based supercomputer facilities, for the digital storage of 3D volumes for research purposes are becoming increasingly more common (e.g. Digital Rocks Portal—www. digitalrocksportal.org; MorphoMuseuM – www.morphomuseum.com; DigiMorph – www.digimorph.org) and their continuing development will enable more and larger archaeological reference datasets to become widely available in the future. The production of animations and 3D printable datasets using microCT data is also effective for media promotion and public outreach and can be employed as engaging tools for teaching purposes.

MicroCT can be integrated with 2D dimensional imaging methods by using a process called registration. This process involves locating (either manually or automatically) the exact three-dimensional location where a 2D image slice occurs in the 3D microCT data and overlaying that 2D image slice to enable direct comparison between different forms of information. The process an be used for registering compositional elemental and mineral mapping techniques such as BSEM (back-scattered scanning electron microscopy) and QEMSCAN with microCT data to identify the presence and distribution of compositional types and link their occurrence to specific greyscales values within 3D datasets (Latham et al., 2008a; b).

## MicroCT applications in Cognate Fields

### Archaeology

Computed tomography has a long history of use in archaeology largely due to the non-destructive nature of the imaging process. This history began with the use of medical scanners to non-destructively image and digitally unwrap mummies and other fragile skeletal remains (Appelboom & Struyven, 1999; Baldock et al., 1994; Cosmacini & Piacentini, 2008; Falke et al., 1987; Hardwood-Nash, 1979; Lynnerup et al., 1997; Sigmund & Minas., 2002). Medical scanners are able to image large samples relatively quickly and produce images of resolutions high enough to answer some types of archaeological and palaeoanthropological questions. The technique’s use in imaging skeletal remains, including both human and faunal, has become almost standard practice (see Zollikofer et al., 1998; Zollikofer & Ponce de Leon, 2005; Weber & Bookstein, 2011). It has been used to successfully identify objects encased within or wrapped with skeletal remains, locate and assess palaeopathologies such as bone fractures or deformities and to analyse general skeletal morphology, to aid in sex and age determination of human remains and species determination of animal remains (Allam et al., 2009; Coutinho Nogueira et al., 2019; Cramer et al., 2018; Johnston et al., 2020; Malgora et al., 2020; McKnight, 2010; Melcher et al., 1997; Panzer et al., 2014; Ru, 2000; Shin et al., 2003). The technique has been used to image a number of high profile archaeological skeletal remains including Oetzi (Gostner et al., 2011; Murphy et al., 2003; Pernter et al., 2007), Tutankhamun (Hawass et al., 2010; Hussein et al., 2013), and Richard III (Appleby et al., 2015). The non-destructive nature of CT scanning has also successfully been used to ‘look’ inside sealed artefacts without having to physically deconstruct or damage objects such as sealed funerary urns (Anderson & Fell, 1995; Harvig et al., 2012; Minozzi et al., 2010) and documents (Applbaum et al. 1995; Stromer et al., 2018).

In the twenty-first century, X-ray microCT has become the most widely used tomography technique for imaging an increasingly diverse range of archaeological materials. Faunal and human bones have been imaged to identify and assess a range of features such as collagen preservation (Beck et al., 2012; Tripp et al., 2018), surface cut marks (Bello et al., 2013a; b; Tuniz et al., 2012), palaeopathological signs of disease and injury (Rühli et al., 2007; Welsh et al., 2020), histological evidence of diagenesis (Booth et al., 2016), and internal anatomical morphologies (Boschin et al., 2015; Bradfield, 2018). The technique has also been used to carry out complex morphological analyses of early hominid fossils (Ponce de Leon & Zollikofer, 1999; Zollikofer and Ponce de Leon, 2005; Zollikofer et al., 2005; Bermúdez De Castro et al., 2011; Holloway et al., 2014; Skinner et al., 2016) and to share that morphological data publicly enabling digital comparative analyses with other hominid specimens (Guipert & Lumley, 2004; Skinner et al., 2013). Other types of archaeological artefacts imaged using microCT scanning include papyrus scrolls (Allegra et al., 2016; Mocella et al., 2015), clay plaster (Bernardini et al., 2018), glass beads (Cheng et al., 2019; Ngan-Tillard et al., 2018), swords (Stelzner, 2016), and coins (Bude & Bigelow, 2020).

### Adaptation from geosciences

MicroCT has long been a common technique for imaging geological and soil samples in the geosciences (Cnudde & Boone, 2013; Ketcham & Carlson, 2001; Taina et al., 2008), leading to the development of analytical techniques that are beginning to be adopted in geoarchaeological and archaeological ceramic studies. Geological studies frequently use microCT imaging to scan rock cores and study a range of properties such as mineral composition, grain size, shape and orientation, pore characterisation and connectedness, and fracture patterns (Bhuiyan et al., 2012; Cnudde et al., 2012; Ersoy et al., 2010; Iovea et al., 2008; Jerram et al., 2009; Ketcham et al., 2010; Rozenbaum, 2011). A number of these properties have since been studied in archaeological ceramics using microCT data to characterize, quantify and visualise the 3D distributions of clay matrices, pores, tempers and other inclusions. These methods have been used to answer archaeological questions relating to vessel manufacturing methods (Coli et al., 2022; Kozatsas et al., 2018; Sanger, 2016) and the provenance of raw materials and vessel types (Bernardini et al., 2019; Kahl & Ramminger, 2012; McKenzie-Clark & Magnussen, 2014).

Soil science studies have used microCT scanning to image intact soil blocks to characterise and quantify solid soil components, physical properties (e.g. porosity and structure) and soil biota without losing 3D contextual information (Cássaro et al., 2017; Chirol et al., 2021; Helliwell et al., 2014; Katuwal et al., 2015; Taina et al., 2008; Voltolini et al., 2017). Traditional soil analysis methods such as 2D thin-section micromorphology can provide higher resolution coverage and additional information (eg. compositional characterization) than microCT, however, it has been shown that microCT is particularly useful complementary technique when used as part of a suite of imaging methods. MicroCT adds three-dimensional contextual information that was previously inaccessible using solely 2D imaging methods and is crucial to understanding dynamic soil processes.

MicroCT analytical techniques previously developed for soil science have been adapted by a number of geoarchaeological studies to image whole soil blocks for virtual investigation (Adderley et al., 2001; Edwards et al., 2017; Huisman et al., 2014; Stelzner et al., 2010; Villagran et al., 2019; Ward & Maksimenko, 2019). Archaeological material types that attenuate X-rays at a higher rate than the soil matrix, such as ceramics, lithics, bone, and shell, can be easily extracted and visualized in three-dimensions for analysis. The 3D microCT data can be used for morphological identification, quantification, or be used as a sampling guide for further analytical methods (i.e. determining the location for 2D thin-section sampling and alerting excavators to the presence of poorly preserved, fragile artefacts during physical excavation). Geoarchaeological case studies to use the technique have so far applied it to the identification of structural features in midden deposits as well as shell, bone, lithic, teeth, scale and gill plate inclusions (Huisman et al., 2014; Ward & Maksimenko, 2019), the characterisation of termite mound materials in archaeological deposits (Villagran et al., 2019), and the visualization of mineral hypo-coatings surrounding faunal burrows in archaeological sediments (Edwards et al., 2017).

### Botany

MicroCT is yet to become a popular imaging technique in the field of botany, largely due to the low attenuating nature of carbon-based organic tissues (Piovesan et al., 2021; Staedler et al., 2013; Stuppy et al., 2003). Staining techniques are oftenrequired before scanning to increase image contrast and differentiate between tissue and cell types (Staedler et al., 2013), or alternatively, phase contrast microCT can be used to detect subtle differences in attenuation (Wilkins et al., 1996). Scans of plants generally must be carried out at low energies with long exposure times, which can increase the occurrence of imaging artefacts. Despite its infrequent use, microCT has been shown to be extremely beneficial in helping to map and quantify the 3D topography of plant structures such as root systems, pore spaces, vasculature, tissue types, and reproductive organs to better understand physiologically processes such as the transport of water, metabolic gases and nutrients throughout entire plant organisms (Brodersen et al., 2011; Dhondt et al., 2010; Ho et al., 2014; Mathers et al., 2018; McElrone et al., 2013; Mendoza et al., 2007; Nugraha et al., 2019; Pajor et al., 2013; Zhao & Takhar, 2017).

Multiple studies have used microCT scanning to map live plant root systems in order to observe and quantify live root-soil interactions without the need for destructive sectioning (Flavel et al., 2017; Maenhout et al., 2019; Mairhofer et al., 2012; Mooney et al., 2011; Phalempin et al., 2021; Teramoto et al., 2020). One of these methods, Rootine (Gao et al., 2019; Phalempin et al., 2021), was originally developed as an adaptation of medical imaging techniques to identify blood vessels based on their tubular shape (Frangi et al., 1998). In these studies, roots are generally identified based on a combination of X-ray attenuation thresholding, shape analysis and object interconnectedness. A number of these root mapping techniques have developed image-processing protocols to, partially (Maenhout et al., 2019; Mairhofer et al., 2012) or fully (Douarre et al., 2018; Gao et al., 2019; Teramoto et al., 2020), automate the processes of identifying and extracting plant roots from within the soil matrix of live plant samples. The trend towards automating the analysis of botanical microCT data is seen in the increasing use of machine and deep learning processes (Earles et al., 2018; Théroux-Rancourt et al., 2020; Soltaninejad et al., 2020; Van De Looverbosch et al., 2021).

Matsushima et al. (2012) used the high-attenuating nature of calcium oxalate crystals to extract druse crystals from surrounding tissues in rose stems. This segmentation process enabled the visualization of druse crystal distribution throughout a botanical organism in three-dimensions for the first time and could prove crucial in forming a better understanding of calcium oxalate formation and function in plants, about which much remains unknown (Franceschi & Nakata, 2005; Nakata, 2003, 2012; Paiva, 2019; Tooulakou et al., 2016). This segmentation method has also proven to be particularly successful at visualizing calcium oxalate crystals in underground storage organs (USOs) to aid in the characterisation of species and identification of archaeological parenchyma fragments (Barron et al., 2022a; Denham et al., 2020).

MicroCT scanning can enable sequential scans of individual organisms at established time intervals and to be registered to create 4D datasets illustrating changes in plant morphology over time (Keyes et al., 2016). This 4D scanning technique has the potential to become revolutionary in understanding how plants develop and respond to various growth conditions because it allows 3D imaging of live samples without the need for destructive mounting procedures.

The three-dimensional nature of microCT data also enables the technique to capture more and/or additional details of reference samples than the traditional method of using 2D histological thin-sections to form botanical reference collections. Currently, there are very few publicly accessible 3D reference collections utilizing microCT data. X-plant (www.X-plant.org), while still in its infancy, is one example of how these collections could be set up and used. The platform currently hosts 41 datasets representing 34 plant species of accessible and downloadable microCT data (MeBioS Postharvest Group, 2022).

### Palaeobotany

MicroCT scanning has been successfully applied to the morphological study of fossilized palaeobotanical remains by scanning already extracted botanical samples and scanning rock fragments and digitally extracting contained botanical fossils. Studies have included analysis of prehistoric plant remains consisting of a wide range of botanical material types including seeds (Smith et al., 2009), fruits (Collinson et al., 2016; Devore et al., 2006; Matsunaga et al., 2019; Smith et al., 2009), flowers (Friis et al., 2014; Moreau et al., 2014), and cones (Karch et al., 2017). MicroCT data has enabled the virtual dissection of extremely rare and fragile specimens to view internal features previously inaccessible using 2D imaging techniques, which may have previously led to the destruction of rare samples. The large quantity of morphological information provided by microCT is particularly important for extracting the maximum amount of information from possibly unique, unidentified specimens to aid in anatomical characterisation and systematic classification (Matsunaga et al., 2019). In the future, microCT may also be used as a way to digitally preserve palaeobotanical samples that are subject to decay over time, or to make damaged samples available for virtual study (Collinson et al., 2016; Devore et al., 2006).

## MicroCT Applications in Archaeobotany

The application of microCT scanning to the study of archaeobotanical remains was almost non-existent ten years ago. A handful of novel studies have recently begun to explore how microCT-based analytical techniques, developed in geology, botany and palaeontology, can be adapted and applied archaeobotanical materials to complement current archaeobotanical techniques and answer archaeobotanical questions. The majority of these studies have concentrated on taxonomic identification of archaeobotanical specimens including wood species used in the manufacture of culturally significant wooden artefacts (Haneca et al., 2012; Kobayashi et al., 2019; Mizuno et al., 2010; Stelzner & Million, 2015; Whitau et al., 2016), fruits extracted from archaeological midden deposits (Calo et al., 2019, 2020), a citrus-like fruit from a funerary offering deposit (Coubray et al., 2010), and a palm droop from the Royal Palace in Jericho (Moricca et al., 2020). All of these studies used microCT scans to visualize different internal features dependent on the type of archaeobotanical material, the diagnostic value of the feature as well as the voxel size of the scan. These features include cell types such as vascular bundles, fibres and parenchyma (Mizuno et al., 2010; Whitau et al., 2016), seed morphologies (Coubray et al., 2010; Moricca et al., 2020), and tissues layers such as the exocarp, mesocarp, and endocarp (Calo et al., 2019, 2020). In some cases, microCT has been able to discount previous identifications, which had been made using only external, 2D imaging methods (Calo et al., 2019; Coubray et al., 2010).

Wolcott et al. (2021) demonstrated how microCT data has the potential to expand upon the application of morphometrical analyses to archaeobotanical samples, an approach that has become increasingly popular for species determination and assessment of domestication status in archaeobotany (Portillo et al., 2020). Wolcott et al. (2021) scanned archaeological and modern *Citrullus* seeds and used the placement of three-dimensional landmarks to compare the two groups in the hopes of identifying the archaeological seeds to *Citrullus* species. While there was not sufficient variation observed to narrow down the identification to species level using a series of landmarks, Wolcott et al. noted that in the future differentiation may still be possible by comparing the complete three-dimensional morphologies of the two layers, the exotesta and endotesta, which they were able to segment out from the microCT data. Comparison of entire 3D surfaces (Frank et al., 2021; Galinsky & Frank, 2014; Gao et al., 2018; Koehl & Hass, 2015) or automating the selection of landmarks (Boyer et al., 2015; Kristensen et al., 2008) may prove useful in the future for those types of archaeobotanical samples where differentiation between possible species using user-selected landmark morphometrics is extremely narrow.

Currently, one of the issues with the identification of anatomical features and identification of archaeobotanical remains using microCT is that the process relies upon comparing microCT images to archaeobotanical and modern reference images taken using 2D image techniques (Calo et al., 2020). Therefore, the comparison with 3D volumes of archaeobotanical samples is currently indirect and requires a degree of subjective interpretation by the observer. This can be solved in time with the development of microCT 3D reference collections of both botanical and archaeobotanical samples. As Calo et al. (2020) notes, microCT datasets can be virtually sliced to produce two-dimensional images “equivalent to light microscopy” for comparison with existing two-dimensional images and previously published descriptions while still retaining its contextual information in three-dimensional space. As Brodersen (2013) has outlined for wood characterisation, the benefits of using both 2D slices and 3D volumes of the same samples are that traditional diagnostic features can be retained in the two-dimensional images while also allowing researchers to access and assess the usefulness of a new range of three-dimensional anatomical features for identifying and analyzing archaeobotanical samples.

A small number of studies have developed new ways to use microCT to answer other types of archaeobotanical questions, particularly relating to the dating and preservation of archaeological wood. Bird et al. (2008) assessed pore size and interconnectedness as well as the distribution of authigenic mineral contamination in both reference and archaeological charcoal samples to better understand how and the extent to which charcoal is altered or degraded in the environment. This method shows potential to aid in determining the reliability of radiocarbon dates taken from charcoal. Stelzner and Million (2015) also successfully used microCT to aid in dating archaeobotanical samples by analyzing tree rings for dendrochronological dating of wood remains in soil blocks. More novel studies such as these, which focus on specific archaeobotanical materials and features are needed in order to understand the possible outcomes, benefits and pitfalls in applying microCT systematically to large numbers of samples and archaeobotanical material types.

### Sexually-propagated cereal and legume crops

MicroCT imaging has been used to assess the domestication status of some of the world’s most understudied, sexually-propagated food crops. The technique can image three-dimensional morphological traits of plant remains associated with the ‘domestication syndrome’ (i.e. abscission scars, grain size, oil content, testa thickness), namely, traits that change over time under cultivation during the domestication period. Certain traits present within cultivated plant populations are thought to have been selected for by humans, both consciously and unconsciously, over long periods of pre-domestication cultivation. Over time continual trait selection, as well as separation from wild plant populations, led to cultivated plant populations evolving to become set as phenotypically and genotypically distinct from their wild ancestors (Fuller, 2007). The expression of traits and their archaeobotanical visibility differ slightly for each species, but the process to assess the domestication status of plant populations always involves an assessment of domestication status for each individual plant specimen, which is then added to proportional counts of types across a botanical assemblage. The proportions of domesticated versus wild types for a given period at a site are then geographically and chronologically compared to other assemblages of the same crop species.

**Fig. 4:**
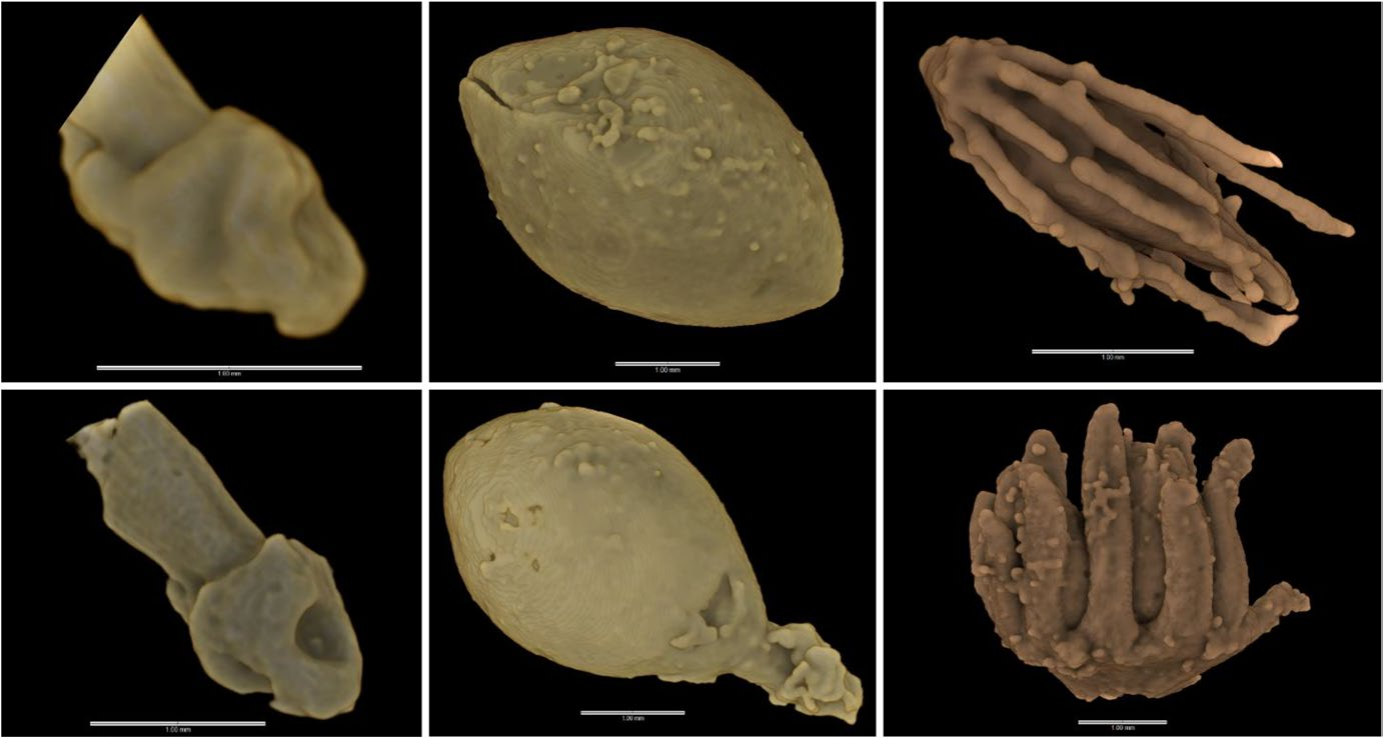
3D renderings of archaeobotanical cereal inclusions from organic-tempered pottery sherds. Top row – Wild types. Bottom row – domesticated types. Left – Rice spikelet bases from Gua Sireh, Borneo. Middle – Sorghum spikelets from KG23, Sudan. Right – Pearl millet spikelets from AZ22 (top) and MK36 (bottom), Mali

The qualitative and quantitative characterisation of domestication syndrome traits has been achieved for multiple cereal crops: rice (Barron et al., 2017, 2020a), sorghum (Barron et al., 2020b) and pearl millet (Fuller et al., 2021)(Fig. 4), and legumes: soybean (Murphy et al., 2019; Zong et al., 2017) and horse gram (Murphy & Fuller, 2017). In each case microCT was able to extract morphological information not accessible using 2D imaging techniques. While 2D imaging techniques such as light microscopy and SEM are often sufficient for assessing external morphologies of stratigraphically extracted archaeological remains, in some cases domestication syndrome traits are not accessible externally and therefore require either destructive sectioning or penetrative imaging. Two-dimensional imaging can provide hints as to the likely 3D morphologies of domestication traits by imaging spikelet abscission scars visible on ceramic surfaces or by visualizing seed coat thickness across a single 2D section, however, studies have shown that for certain domestication traits or certain types of archaeobotanical evidence microCT is the most robust and efficient way to assess domestication status (Barron & Denham, 2018; Barron et al., 2020a; b; Murphy & Fuller, 2017; Murphy et al., 2019; Zong et al., 2017).

Barron et al. (2017, 2020a, b) have demonstrated that microCT scanning can be used to visualize archaeobotanical remains used as organic temper and preserved within archaeological pottery sherds, as well as to determine the domestication status of included cereal spikelets. While 2D surface impression analysis has produced some success in identifying and assessing organic inclusions in the past (Barron et al., 2017; McClatchie & Fuller, 2014; Winchell et al., 2017), the use of microCT imaging provides much greater analytical coverage and therefore is substantially more effective and efficient at extracting archaeobotanical information from pottery sherds (Barron et al. 2020a). This technique has so far been utilized to study rice in sherds from Vietnam (Barron & Denham, 2018; Barron et al., 2017) and Borneo (Barron et al., 2020a), sorghum in sherds from Sudan (Barron et al., 2020b) and pearl millet in sherds from Mali (Fuller et al., 2021). In all these studies, cereal spikelets could be identified in and segmented from the microCT data in order to visualize individual spikelets in three-dimensions for qualitative analysis of abscission scar morphology, which can then be compiled for quantitative assessment of archaeobotanical assemblages.

In each case study, the amount of archaeobotanical evidence available to address, globally significant archaeological questions has been significantly increased. MicroCT has granted access to new archaeobotanical assemblages present in “old” (previously excavated) materials from regions of relatively poor archaeobotanical coverage, where traditional archaeobotanical techniques have not been routinely applied (Castillo & Fuller, 2010; Winchell et al., 2018). For example archaeobotanical sampling has been inconsistently used at archaeological sites across Southeast Asia, meaning that we have very poor understanding of the timing and mechanisms of diffusion for rice farming into the region (Denham, 2013; Donohue & Denham, 2010; Paz, 1999, 2002). This lack of evidence is particularly pronounced in Island Southeast Asia, where theories regarding the introduction of rice farming to a locale have sometimes been dependent on merely the presence of a single rice grain lodged in the surface of a single pottery sherd from a single site (Barron et al., 2020a; Bellwood et al., 1992). MicroCT imaging demonstrated that no other rice remains were present in the pottery sherd, thereby calling into question current models of diffusion dependent on the single, likely wild, rice grain.

Ideally, assertions regarding the timing and trajectories of domestication episodes are based on the analysis of large archaeobotanical assemblages from numerous archaeological sites, of high geographical and chronological diversity (Fuller, 2007; Fuller et al., 2012, 2014; Purugganan & Fuller, 2011). The assemblages can then be collated and compared to form a domestication curve to form a better overall picture of the timing and trajectories specific to each crop or domestication event. Robust domestication curves have been developed for certain crop species such as wheat (Allaby et al., 2017), maize (Kistler et al., 2018) and rice (Fuller et al., 2016), consisting of the analysis of thousands of cereal spikelets from increasingly large numbers of sites. Unfortunately, robust domestication curves have not been established for all sexually propagated crops due to the relative scarcity of available archaeobotanical specimens. Some of these understudied crops have been the focus of microCT studies including sorghum and pearl millet, which were previously assumed to have originated in Sahelian Africa despite some of the earliest evidence of their domestication being located in India (Kahlheber & Neumann, 2007; Winchell et al., 2018).

MicroCT was shown to be so useful at identifying sorghum spikelets in ceramics from the site of KG23 in Sudan that it recovered spikelets at a rate of over eleven times the rate of surface impression analysis (Barron et al., 2020b). The three-dimensional technique was able to identify 82 spikelets in 12 sherds, equating to a rate of 6.83 spikelets per sherd, compared with the two-dimensional technique of surface impression analysis that identified 51 spikelets on 91 sherds at a rate of only 0.56 spikelets per sherd. The addition of these 82 new sorghum spikelets to the previously studied 51 surface spikelets makes the combined KG23 botanical assemblage the biggest pre-domestication sorghum assemblage known to date. Therefore, this one study of only a handful of sherds has demonstrated the added efficiency and increase in archaeobotanical evidence that can be achieved by imaging organic-tempered pottery using microCT.

Murphy and Fuller (2017) and Zong et al. (2017) have similarly used microCT to image and assess domestication traits in the legume species of soybean and horse gram respectively. Murphy et al. used synchrotron microCT to image and measure seed coat thickness for a selection of archaeological horse gram specimens from India dating from between 2000 and 1200 BC. Seed coat thinning is thought to be linked to cultivation selection due to the resultant reduction in seed dormancy. Murphy et al. were able to show that horse gram seed coats became thinner over the time period in a stepped progression from thick (older than 1700 BC) to semi-thin (1800 BC to 1300 BC) and from semi-thin to thin (later than 1300 BC). This was the first time seed coat thinning was demonstrated in a legume species from the analysis of archaeological remains and contradicted assumptions that domesticated forms were derived from low dormancy wild populations. They also found there to be considerable variation in seed coat thickness within individual seeds as well as between different seeds. This highlights the importance of the 3D coverage provided by microCT to enable measurements to be taken at multiple points in order to attain the most accurate morphometric data.

Zong et al. (2017) used microCT to investigate the oil content in archaeological soybeans as oil content has been shown to be higher in modern domesticated varieties than wild plants. They argue that higher oil content in seeds creates small internal holes whereas higher protein content creates large internal holes. By imaging soybeans from four archaeological sites in China, dating from between 7500 cal BP to 4000 cal BP, Zong et al. found that the occurrence of small holes gradually increased over time showing that soybeans with higher oil content were selected for under cultivation. Murphy et al. (2019), however, have suggested that the presence of holes in archaeobotanical soybeans and their size could be affected by pre-depositional charring and/or preservation conditions. These studies suggest further work is needed, including the analysis of experimentally charred seeds using microCT imaging, before the cavities in soybeans can be conclusively linked to oil content and, by extension, human-mediated selection under cultivation.

All of these methods discussed, which use microCT to assess domestication status, have relied on the technique’s abilities to image internal morphologies and to visualize those morphologies in three-dimensions. While these techniques are in their initial stages of development, they have opened up new avenues of research in geographic regions and for sexually propagated crop species that are significantly underrepresented in the archaeobotanical literature.

### Asexually-propagated USOs

The archaeological study of asexually propagated crop species is limited in comparison to sexually propagated crops, particularly cereals (Denham et al., 2020). There are a number of likely reasons why this is the case including their lower archaeological visibility due to the lack of taphonomically robust forms of evidence (i.e. seeds), the complexity of identification methods, as well as lower archaeobotanical sampling in the regions from which many of these crops originate. Despite these issues, the consumption, management and cultivation of asexually propagated crop species have been shown to be extremely ancient practices (Clarkson et al., 2017; Denham et al., 2003; Florin et al., 2020; Wadley et al., 2020), which have been overlooked archaeologically for many decades creating an incomplete view of ancient subsistence strategies and likely inflating the importance of seed-based agriculture for populations reliant on mixed subsistence practices.

A suite of new archaeobotanical techniques has been developed in recent decades to extract, identify and analyse new types of archaeobotanical evidence such as phytoliths (Piperno, 2006), starch (Barton & Torrence, 2006) and archaeological parenchyma (Hather, 2000) produced by asexually propagated plants. These forms of evidence are less archaeologically visible than the macro-remains of sexually-propagated plants and their identification methods can be inconclusive for taxonomic characterisation when used in isolation. Therefore, there is a need to more regularly employ a multi-proxy approach in archaeobotany to increase both the quality and quantity of archaeobotanical data (García-Granero et al., 2015).

One type of evidence and investigation technique, which remains extremely rare in the literature is the study of preserved fragments of parenchyma tissues from the fleshy, starchy organs of roots, tuber and corms (Hather, 1991, 1993, 1994, 2000; Kubiak-Martens, 2016; Oliveira, 2008, 2012; Paz, 2001, 2005; Ussher, 2015). These are often known collectively as underground storage organs (USOs), or in archaeobotanical contexts archaeological parenchyma, and include some of the world’s most economically important food crops such as sweet potato, potato, manioc, taro and yams. A robust technique to characterize fragments of these archaeological parenchyma tissues was developed by Jon Hather in the 1990s (Hather, 1988, 1991, 1993, 1994; 2000; Hather & Kirch, 1991) but its potential to expand archaeobotanical knowledge has yet to be fully exploited (Hather & Mason, 2009; Kubiak-Martens, 2016).

As these USO organs are composed of almost entirely undifferentiated starch-storing parenchyma cells and exhibit high levels of intraspecies phenotypic plasticity, Hather’s identification method requires the characterisation of a complex range of anatomical and morphological features for each fragment of archaeological parenchymatous tissue in order to secure a likely taxonomic identification (Hather, 2000; Paz, 2001). These features include surface morphology, tissue organization, vascular bundle arrangement and distribution, calcium oxalate crystals, and patterns of charring or vesicularisation. Some features are considered more diagnostic due to wide interspecific and narrow intraspecific variety than others that present wide intraspecific variability that overlaps significantly with other species and families.

One factor restricting the widespread use of Hather’s identification method is that to characterize diagnostic USO features requires the development of extensive and accessible reference collections, which capture the broad range of morphological variability within and between species. The analytical coverage provided by microCT would be more effective at capturing the range of variability than the 2D sampling methods used to date (light microscopy and SEM), which are incapable of illustrating all of the diagnostic features of a single USO sample in a single or small handful of slices.

The ability of microCT to capture those diagnostic features identified by Hather has to date been demonstrated by only three studies, Pritchard et al. (2019) and Barron et al., (2022a & b). Pritchard et al. (2019) used SEM and light microcopy images of yam (*Dioscorea nummalaria*) and sweet potato (*Ipomoea batatas*) reference samples to compare to microCT images of archaeological parenchyma fragments from Kuk Swamp, Papua New Guinea. Similarities in diagnostic features such as vascular bundle arrangement and distribution were found in the reference images for sweet potato to the microCT data for two archaeological parenchyma fragments, thus supporting previous identifications of sweet potato (Lewis et al., 2016). While these results are archaeobotanically robust, the security of taxonomic determination could only be further increased by the scanning of a wide range of relevant reference species in three-dimensions for direct comparison.

Barron et al. (2022a) in their study of five of the most significant asexually-propagated crops in Southeast Asia and the Pacific: taro (*Colocasia esculenta)*, sweet potato (*Ipomoea batatas),* giant taro (*Alocosia macrorrhiza*), and two yams species, *Dioscorea esculenta* and *Dioscorea alata,* have taken the first steps towards compiling an archaeological reference collection of archaeological parenchyma species using microCT. The five species study scanned Hather’s own charred reference specimens and used his diagnostic criteria as a guide to differentiation of plant families and species but was also generally focused on qualitative pattern recognition between datasets, as it was assumed that by imaging these organs in three-dimensions for the first time new and different features of characterisation would be also captured. It is also likely that complex patterns of differentiation exist in parenchymatous organs that are not apparent to a human analyst but could be identified with the application of deep learning processes as discussed below.

One significant factor noted by Barron et al. (2022a) was that variation of features such as cell size and shape, damage from charring and the distinction between tissue layers was incredibly high even within individual USO fragments. This variation showed that imaging of whole archaeological samples is incredibly important as the characterisation of a USO based on a single 2D image could be extremely misleading as to the nature of the whole organ. Another feature of the reference samples scanned is that there is a much wider range of calcium oxalate crystal-types in samples of the same species than has been documented previously. Samples of taro and sweet potato displayed a variety of crystal types and sizes that suggested some oxalate crystal attributes could provide more information regarding the environmental growth conditions of a USO organ than species identification and so a combination of characteristics must be taken into consideration, including crystal size, shape, frequency and distribution.

The reference collection being compiled by Barron et al. (2022a) is planned to be expanded to include other species of USO species significant to the Southeast Asia, Pacific and Oceania regions as well as to be made publically available online to aid in more robust and frequent archaeological parenchyma identifications as well as in the hopes of making the analysis of archaeological parenchyma more accessible.. By making 3D volumes of reference and archaeological samples available online, this will enable other researchers to interrogate those datasets, and any interpretations that may be the result of subjective interpretation by the observer (Baveye, et al., 2010) as well as facilitate intersample comparability. Barron et al.’s (2022b) subsequent successful use of microCT reference datasets to identify archaeological parenchyma fragments from Nombe, Papua New Guinea, as sweet potato sp. shows that the identification of archaeological parenchyma is a complex process that can benefit from the 3D morphological complexity provided by microCT scanning of relelvant reference specimens.

## Future Applications

The potential for using microCT imaging in archaeobotany is just beginning to be explored. MicroCT scanning technology and 3D image processing capabilities continue to improve, the technique’s benefits to the field could increase exponentially. While a number of novel archaeobotanical applications have already been developed as outlined above, their application to the analysis of larger quantities of samples will ultimately demonstrate the technique’s potential to transform traditional archaeobotanical approaches by the quantity, quality and robustness of results. Subsequently, the technique could aid in reforming and reframing current archaeobotanical questions and assumptions by significantly expanding, what are in some fields, incredibly small pools of available evidence.

The two forms of information that microCT has contributed to the archaeobotanical studies outlined above are: internal morphologies and secure quantitative, and specifically volumetric, measurements. Therefore, it has been shown that microCT can prove useful in answering archaeobotanical questions reliant on these two kinds of information. The technique could also provide useful in situations where small quantities of fragile or valuable samples exist from which it is important to obtain maximum amounts of information and/or those samples are intended to be used for other destructive sampling methods if it is deemed appropriate. The non-destructive nature of microCT means it can be used for morphological imaging before the application of subsequent analytical methods, in a similar way to how it has been applied in a number of geoarchaeological studies, as a complementary technique to more traditional methods, as one component in a suite of analytical approaches (Calo et al., 2020; Edwards et al., 2017; Huisman et al., 2014; Machado et al., 2017).

The risk with 3D imaging is the temptation to produce large amounts of three-dimensional, high-resolution data under the assumption that this will produce equivalently significant results. However, the sheer amount of data produced by microCT can mean that it becomes difficult for even specialist individuals to manually sort through and extract the maximum amount of relevant and/or useful information contained in a single dataset (Barron et al., 2020a). Additionally, these large datasets are currently difficult to store, especially if large reference collections of even tens of samples are compiled, which could equate to many terabytes of data (see Rowe & Frank, 2011 for overview of problems relating to 3D data storage). For example, the 30 datasets analysed by Barron et al. (2022a) representing just five USO species added up to a combined total of 3 terabytes of data. Therefore, using current user-driven processing methods, unless significant time and expertise can be allocated to manual data analysis, it may in many instances be difficult to justify the economic costs of microCT, which for individual samples are much greater than light microscopy or SEM. The economic costs can be made much more affordable by the scanning of many archaeobotanical samples in a single scan and, in the future, the time and energy costs could become much more reasonable with the automation of some or all of the data processing stages.

Currently, the dominant trend in 3D data processing is towards the automation of segmentation and analysis processes in order to exploit the ever increasing amounts of data that high-resolution 3D imaging produces. The automation of segmenting microCT datasets can be achieved relatively easily, depending on the sample type and specific research questions, by establishing a series of algorithmic filters through which data is progressively processed to extract all of the specified relevant information. Simple filters include those discussed above that are widely used to segment geological and botanical samples such greyscale values or gradient, size, shape, or connectedness. These series of filters can then be applied to batches of microCT scans to process large amounts of data extremely quickly. Herremans et al. (2015) developed an automated segmentation protocol to segment void and cell networks of fruit parenchyma tissues and identified significant differences between genotypes. Segmentation protocols using the same basic concept could be developed in the future to aid in the characterisation and identification of archaeobotanical specimens.

**Fig. 5:**
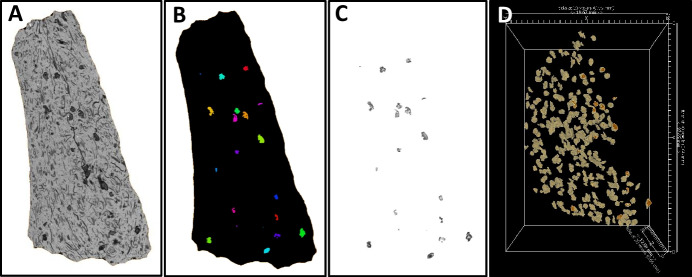
Segmentation process to separate rice spikelet bases from tomographic data of whole rice-tempered pottery sherd. A. 2D x-ray slice of sherd showing rice temper (lower x-ray attenuation) in black. B. Isolation and labelling of each rice spikelet base based on x-ray attenuation, size and shape. C. 2D slice of isolated rice spikelet bases with x-ray attenuation values. D. 3D rendering of rice spikelet bases from slice A-C (closest to camera) plus all spikelets bases in subsequent slices

The process of automating data segmentation of rice spikelet bases from a pottery sherd has been trialed by the author on a rice-tempered sherd from Gua Sireh that was previously analysed by Barron et al. (2020a). In the previous study, only 50 rice spikelet bases were analysed due to the sheer number of spikelet bases found to be contained within the sherd and the long processing times required for each individual spikelet base. While it is relatively easy to segment out all of the organic inclusions in a sherd using basic greyscale thresholding, the segmented organic fraction will inevitably include large amounts of undiagnostic chaff, including husk, leaf and stem fragments, as the organic materials used for temper would have included all the waste products leftover from the rice processing stages (see Harvey & Fuller, 2005; Fig. 3). Therefore, following greyscale thresholding a series of data filters were needed to label each isolated inclusion and then to exclude as much chaff material as possible while selecting the spikelet bases. The most effective way to achieve this was to filter the data by shape, selecting only the roughly spherical spikelet bases (not the rod-like or more irregular chaff fragments), and by size, eliminating those inclusions with voxels volumes significantly larger or small than the average spikelet base morphology. The visual results of this segmentation process are shown in Fig. 5 including a microCT greyscale slice (5A), the same slice with labelled inclusions after segmentation by size and shape (5B), the same slice with greyscale values of segmented inclusions (5C), and a 3D rendering of segmented inclusions, most of which represent rice spikelet bases (5D).

The difficulties of working with archaeological data became apparent during this segmentation experiment and the major barriers to automation were identified. The lack of control over archaeological data, its lack of structure, and possible taphonomic alteration make it incredibly difficult to standardize archaeological imaging protocols. This was evident when attempting to filter the chaff data by shape and size with the overlapping nature of the chaff material resulting in many individual spikelet bases not able to be isolated and, therefore, excluded from subsequent analyses. There were also many chaff pieces that were similar enough to spikelet bases in shape and size that they were not excluded from the segmentation. These issues are not unique to archaeological data, however, they add levels of complexity that do not exist in other fields, such as industrial engineering and manufacturing, and will require more complex processing than has yet been attempted in the field of archaeobotany.

### Machine learning

The Gua Sireh rice spikelet base segmentation outlined above was undertaken in order to prepare the dataset to be used as a baseline teaching dataset in the development of a neural network able to automatically identify wild and domesticated rice spikelet bases. Neural networks are essentially sets of algorithms that utilize machine learning to numerically recognize patterns in order to cluster and label data. To prepare the Gua Sireh segmented dataset for training a neural network, half of the individual labelled inclusions were visualized and designated as either ‘a spikelet base’ or ‘not a spikelet base’, the domestication status of the spikelet bases was qualitatively assessed by two specialist archaeobotanists and then the characterisation of each inclusion was compiled.

In the future, this information will be the initial input layer of a neural network to enable distinction between spikelet bases and non-spikelet bases as well as domesticated and wild abscission scar morphologies. The neural network will then be tested against the other half of the inclusions in the Gua Sireh dataset and the results assessed by manual specialist analysis. Multiple iterations of this process are likely to be required before high levels of accuracy can be achieved. The development of this new neural network is ongoing and the initial results appear promising. If the results are successful for the Gua Sireh rice spikelet bases, the neural network could be used to analyse scans of multiple pottery sherds with hundreds of rice spikelet inclusions almost instantaneously. However, to analyse spikelets of other cereal species used as temper, new neural networks would need to be established to recognize the distinct domestication syndrome traits for each cereal crop species.

In archaeobotany, an attempt has previously been made by Wilson et al. (2010) to use supervised learning algorithms to automate the process of taxonomically identifying starch grains from nine crop species. To do so they measured a series of variables relating to the shape, size, curvature and polarization cross of starch grains from botanical reference specimens, to produce the initial input layer of data used to establish the starch grain output labels and their characteristics. The measurements of taxonomically known starch grains were then fed into the network as a test dataset in order to assess the network’s ability to classify each starch grain based on the characteristics of the reference species. The rates of successfully identifying unlabeled starch grains were extremely variable, falling between 14–85% depending on the species. These results illustrate that some species of starch grains possess significant morphological overlap and, using current methods, cannot yet be robustly identified to species level. Significantly, the authors noted that the results were limited by the fact that they only utilized 2D light-microscopy images meaning that the 3D starch grains would have all been measured in differing orientations. This additional variation is a problem that three-dimensional imaging methods, such as microCT, can be used to overcome.

Kobayashi et al., (2015, 2019) have used CT data of wood reference samples to develop a series of feature extractor and classifier algorithms to automate the identification of wood species of valuable cultural artefacts. Their input reference data consisted of 10 wood samples from 8 of the most commonly used wood species for Japanese sculptures. While their base datasets were three-dimensional CT scans, the images selected for feature extraction and classification consisted of 40 two-dimensional slices from each 3D dataset precisely cropped to provide clear transverse sections. To date, Kobayashi et al.’s method has shown promising results, having identified 10 wood samples accurately, however, more species and more samples of each species will ensure more robust results in the future.

### Deep learning

Deep learning networks could also be utilised in the future to tackle particularly complex archaeobotanical analyses, like the identification of USO species of archaeological parenchyma, both efficiently and reliably. Deep-learning is a form of machine-learning that can automatically extract features from raw data as that data is processed through multiple hidden layers of algorithmic filtration before classification. Essentially, deep learning has the ability to determine the appropriate parameters for classification independently, thus creating a classification process that is entirely automated from end-to-end (LeCun et al., 2015; Schmidhuber, 2015). Deep learning algorithms are already revolutionizing research in fields such as biometrics (Sundararajan & Woodard, 2018), visual, audio and text classification (Minaee et al., 2021), social network analysis (Pouyanfar et al., 2019) and medicine (Wang et al., 2019), including to accurately diagnose Covid-19 in lung CT scans (Wang et al., 2021).

Most importantly, deep learning processes benefit from large data inputs, as the more data available for analysis creates more robust networks. Therefore, deep learning is a way of exploiting the increasingly large size of 3D datasets created by microCT scanning and extracting the maximum amount of diagnostic information, some of which may not even be evident to a human specialist through visual inspection. In archaeobotany, a deep-learning network could prove particularly useful in differentiating intraspecies and interspecies variation in USOs considering it is already difficult for a human user to quantify the range of variation of some features across single samples or individual species. The technique could also be incredibly useful in efforts to establish and describe a set (or sets) of traits that combine to form a domestication syndrome in these asexually-propagated USO crops by compiling microCT data of a range of ancient and modern samples and determining qualitative and/or quantitative differences between the two.

## Conclusions

Despite computed tomography’s long history of use in archaeology, the potential benefits of utilizing the technique for archaeobotanical research has only begun to be explored in the last ten years. The advent of microCT, with its ever increasing image resolution, coupled with advances in computational processing power mean that microCT’s research capabilities are constantly expanding. A range of microCT visualization and analytical methodologies have been developed in related fields such as archaeology, geosciences, botany and palaeobotany, which could be applied, or adapted for application to a range of archaeobotanical materials. In addition, a handful of recent archaeobotanical studies have presented novel quantitative and qualitative microCT methodologies and identified potential new forms of evidence, which can be extracted from previously excavated materials. These novel methodologies have already been shown to have expanded the extremely limited knowledge we have of the origins and spread of some of the world’s most economically important food crops, including a range of sexually-propagated cereal and legume species, and aided in the secure identification of remains of some of the most understudied crops, asexually-propagated underground storage organs. The amount of information produced by microCT imaging may not be required for all archaeobotanical studies and, currently, scanning costs and analysis times are high. However, the rapid development of machine- and deep-learning networks could, in the future, enable extremely fast and efficient analysis of large numbers of samples and/or extremely robust classifications of complex forms of evidence, leading to more widespread use of the technique in the field of archaeobotany.

## Data Availability

CT data used for figures is available from the author upon request.
